# Aqueous Extract of *Brassica rapa* L.’s Impact on Modulating Exercise-Induced Fatigue via Gut–Muscle Axis

**DOI:** 10.3390/nu15224737

**Published:** 2023-11-09

**Authors:** Cheng Wang, Hongkang Zhu, Yuliang Cheng, Yahui Guo, Yong Zhao, He Qian

**Affiliations:** 1 School of Food Science and Technology, Jiangnan University, No.1800 Lihu Avenue, Wuxi 214122, China; 6200113209@stu.jiangnan.edu.cn (C.W.); 7210112118@stu.jiangnan.edu.cn (H.Z.); ylcheng@jiangnan.edu.cn (Y.C.); guoyahui@jiangnan.edu.cn (Y.G.); 2 Collaborative Innovation Center of Food Safety and Quality Control in Jiangsu Province, Jiangnan University, Wuxi 214122, China; 3 Department of Thoracic Surgery, Affiliated Hospital of Jiangnan University, Wuxi 214000, China

**Keywords:** *Brassica rapa* L., exercise-induced fatigue, energy metabolism, inflammatory response, intestinal microbiome, gut–muscle axis

## Abstract

Exercise-induced fatigue is a common physiological response to prolonged physical activity, often associated with changes in gut microbiota and metabolic responses. This study investigates the potential role of *Brassica rapa* L. in modulating these responses. Using an animal model subjected to chronic exercise-induced stress, we explored the effects of *Brassica rapa* L. on fatigue-related biomarkers, energy metabolism genes, inflammatory responses, intestinal integrity, and gut microbiota composition. Our findings revealed that *Brassica rapa* L. exhibits significant antioxidant activity and effectively modulates physiological responses to fatigue. It influences gene expression related to the tricarboxylic acid (TCA) cycle in muscle tissue through the AMPK/PGC-1α/TFAM signaling pathway. Furthermore, *Brassica rapa* L. has been found to alleviate inflammation by inhibiting lipopolysaccharide (LPS) infection and suppressing the activation of the NF-κB pathway. It also maintains intestinal integrity and controls Gram-negative bacterial growth. A correlation analysis identified several pathogenic bacteria linked with inflammation and energy metabolism, as well as beneficial probiotic bacteria associated with improved energy metabolism and reduced inflammation. These findings underscore *Brassica rapa* L.’s potential for managing prolonged exercise-induced fatigue, paving the way for future therapeutic applications. The results highlight its impact on gut microbiota modulation and its role in nutrition science and sports medicine.

## 1. Introduction

*Brassica rapa* L., commonly known as turnip, is a medicinal and edible plant primarily found in plateau areas. It was planted on Lhasa, Tibet, situated at an average altitude of 3800 m, with an average annual temperature of about 8 °C and an annual precipitation of about 500 mm. Due to the unique high-altitude conditions, including low oxygen levels, intense ultraviolet radiation, and extreme climate, certain environmental adaptation gene expressions can be triggered in plateau-dwelling plants. This genetic adaptation can lead to metabolic changes in plants, ultimately resulting in distinct pharmacological effects such as anti-oxidation, anti-inflammation, and immune optimization, as well as physiological activities such as hepatoprotection, tumor inhibition, and neuron protection [1,2]. For instance, according to the Tibetan medicinal dictionary, *Rhodiola rosea* L. roots have traditionally been used to alleviate pneumonia, tracheitis, and weakness. Current pharmacological research suggests that *Rhodiola*, particularly its component Salidroside, can effectively extend its anti-fatigue effects [3,4]. Furthermore, *Rheum palmatum* L. roots have been traditionally used for the treatment of high fever, profuse sweating, constipation, and abdominal pain. Recent research has highlighted that the anthraquinones present in this plant may have the most promising effects, particularly in modulating fatigue [5]. In addition, *Brassica rapa* L. has been cultivated by Tibetans for a long time to relieve various physical discomforts, such as fatigue and hypoxia [6]. Previous studies have investigated the bioactive compounds in *Brassica rapa* L., such as saponins, polysaccharides, and glucosides, and their potential immune-boosting and metabolism-optimizing properties [7]. Our recent research has identified numerous constituents in the aqueous extract of *Brassica rapa* L. (AEB), including organic acids, esters, thioglycosides, glycosides, flavonoids, polyphenols, etc. [8]. We have also assessed its potential in reducing acute exercise-induced fatigue and its muscle-protective properties. However, this was an initial exploration; the underlying mechanisms and the consistency of these effects warrant further investigation.

Fatigue can be characterized as a subjective sensation of discomfort, manifesting as an intense feeling of weariness and depletion [9]. It typically arises due to various physiological, pathological, and psychological imbalances within organisms [10]. Currently, a significant number of individuals are experiencing fatigue-related disorders due to stress from work and life, which can be characterized as chronic stress-induced fatigue. Therefore, the development of drugs to alleviate these chronic fatigue symptoms is of substantial importance. During prolonged, high-intensity exercise, the body’s internal environment is disrupted due to energy expenditure and metabolite accumulation. This can potentially result in muscle soreness and spasms, as well as dyskinesia [11]. Recent studies have confirmed that disorders in the gut microbiome can manifest as symptoms of fatigue [12,13]. Emerging research has highlighted the role of the intestinal microbiome as a critical regulator in alleviating fatigue-related stressors, including oxidative stress, inflammatory responses, and intestinal damage [12]. Actually, the gut–muscle axis refers to the bidirectional communication between the gut and skeletal muscles, which can influence muscle function and overall health [14].

We have discovered that *Brassica rapa* L. extract can optimize probiotics in vitro fermentation, suggesting its potential function in gut health [15]. These findings provide a theoretical basis for the deep investigation of the bioactive mechanism of *Brassica rapa* L. through its influence on the gut–muscle axis. In this study, we aim to investigate the mechanisms by which *Brassica rapa* L. influences fatigue during prolonged exercise. Initially, we established a mouse model of prolonged exercise to simulate chronic physical stress. Subsequently, we assessed fatigue-related biomarkers and further investigated the energy metabolism process in muscle tissue, as well as the inflammatory response in both muscle and colon tissues. Finally, we employed 16S rDNA analysis to examine the composition of the intestinal microbiome. Correlation analyses were conducted to gain insight into how these microbiomes affect peripheral parameters and to measure the effects of *Brassica rapa* L. on the gut–muscle axis and how they modulate fatigue.

## 2. Materials and Methods

### 2.1. Materials

*Brassica rapa* L. roots were purchased from Tibet. The rest of the chemical reagents used in this experiment were analytically pure.

### 2.2. Preparation and Quantification of Samples

#### 2.2.1. Preparation of Aqueous *Brassica rapa* L. Extract

We obtained the aqueous extract of *Brassica rapa* L., hereafter referred to as AEB, through the following process: Fresh *Brassica rapa* L. specimens were first washed and sectioned before being oven-dried at 60 °C. The dehydrated samples were procured. These dehydrated samples were then subjected to two rounds of extraction in boiling water for one hour each, maintaining a solid-to-liquid ratio of 1:10 (*w*/*v*). Afterward, the mixtures were centrifuged at 4000 rpm for ten minutes and filtered to eliminate any residual matter. The resulting extract was lyophilized in a vacuum freeze dryer at −70 °C. Ultimately, the lyophilized powder was stored in vacuum-sealed, light-resistant glass containers at room temperature for future research.

#### 2.2.2. Quantification of Constituents

According to the standard methods provided, the main constituents were determined. The Kjeldahl method was utilized for protein quantification (GB 5009.5-2016 [16]). The total polyphenols were determined using aluminum nitrate (T/AHFIA 005-2018 [17]). The total flavonoids were analyzed with the folin-phenol reagent (referred GB/T 20574-2006 [18]). The phenol-sulfuric acid method was employed to measure the total sugar (referred NY/T 2332-2013 [19]). The total saponin content was detected through a reaction with vanillin under acidic conditions (DB22/T 1668-2012 [20]). Alkali hydrolysis was applied to quantify the total fat content (referred GB 5009.168-2016 [21]). All of these measurements were confirmed using spectrophotometry. The fiber content was quantified using the filtration method (referred GB/T 5009.10-2003 [22]). Following acid hydrolysis, amino acids were subsequently analyzed via HPLC (referred GB 5009.124-2016 [23]).

### 2.3. Animal Treatment

#### 2.3.1. Experimental Animals

Thirty-two male Swiss mice (8 weeks old; 39~42 g) were purchased from Vital River Laboratory Animal Technology Co., Ltd. (Beijing, China). All procedures involving these experimental animals were approved by the Ethics Committee of the Experimental Animal Center of Jiangnan University (JN.No20211130k0341225). The mice were acclimated to laboratory conditions for 5 days and housed in a controlled environment at 22 ± 2 °C, with 55 ± 15% relative humidity and a 12 h light/dark cycle. They had free access to water and food throughout the study. The feed was purchased from Cooperative Medical Biological Engineering Co., Ltd. in Suzhou, China. Details of the diet which was utilized can be found in the Supplementary Materials.

#### 2.3.2. Experimental Groups and Treatments

**Figure 1:**
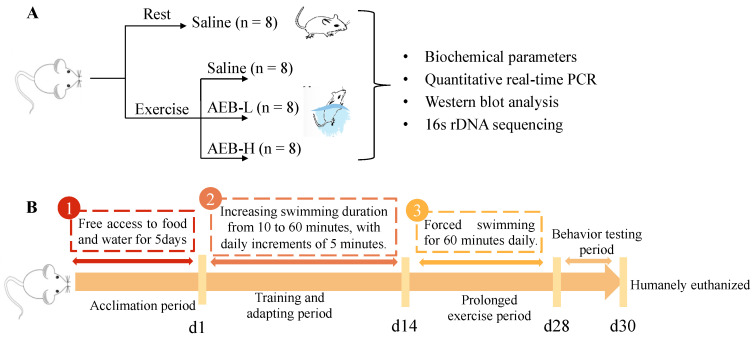
The experiment protocol on mice. (**A**) Experimental groups and treatments, (**B**) Exercise protocol.

The daily recommended intake of *Brassica rapa* L. for humans is approximately 6 g (dehydrated sample), and the average adult body weight is considered to be 60 kg. Thus, we adjusted this dosage for mice by a factor of ten due to differences in metabolism. Therefore, after calculations, it becomes evident that the maximum daily recommended dose for mice equates to about 1 g/kg body weight. The AEB powder was diluted with deionized water to 50 g/L (low dose) and 100 g/L (high dose) concentration for further gavage. After the acclimation period, the mice were randomly divided into four groups, as shown in Figure 1A (*n* = 8):

- Control group was gavaged with saline (Con);
- Exercise group was subjected to swimming and gavaged with saline (Ex);
- Exercise group was subjected to swimming and gavaged with AEB powder at a low dose of 0.5 g/kg body weight (AEB-L);
- Exercise group was subjected to swimming and gavaged with AEB powder at a high dose of 1 g/kg body weight (AEB-H).

The Con group did not participate in any swimming activity, but were handled daily, similarly to other groups, to mimic the disturbance caused by the experiment.

#### 2.3.3. Exercise Protocol

As depicted in Figure 1B, for 5 days post-acclimation, the mice in the Ex, AEB-L, and AEB-H groups were subjected to a swimming exercise regimen designed to induce fatigue. This protocol was implemented daily for four weeks, simultaneously with their respective treatments.

The exercise involved forced swimming in a tank filled with water (30 cm depth, maintained at 25 ± 2 °C). On the first day of the regimen, each mouse was made to train for 10 min. Thereafter, we increased the swimming duration by 5 min daily until it reached a steady state of one hour on Day 14. This served as both a training and adaptation period for the mice in preparation for more intensive exercise. After this adaptation phase, we maintained this one-hour swimming duration for them throughout the remainder of the experimental period [24]. To ensure active swimming and to prevent floating during each session, we immersed the mice up to a level where their hind limbs could not touch the bottom of the tank.

All sessions were conducted from 9 a.m. to 2 p.m. each day to avoid variations due to circadian rhythms. This timing also corresponds with the period of least variation in the mice’s aerobic metabolism, further reducing potential confounding factors. After each session, the mice were towel-dried and returned back into their cages with free access to food and water.

#### 2.3.4. Sample Collection

On the 28th day of exercising, fecal samples were obtained from all mice before the swimming exercise session. On the 30th day, after completing the swimming exercise session, all mice were humanely euthanized following institutional guidelines. The dissection process was carried out promptly to obtain various samples. Blood samples were collected immediately after euthanasia and placed in appropriate tubes for further analysis. The collected blood samples were then centrifuged at 4000 rpm for 15 min at a temperature of 4 °C to separate serum from blood. Tissue samples from the liver, colon, and gastrocnemius muscle were also harvested during dissection. To ensure preservation prior to subsequent stages of analysis, all collected samples were stored at −70 °C.

### 2.4. Biomarkers Determination

Blood glucose (BG) levels were determined using a Roche Glucometer (Roche Diabetes Care GmbH, Basel, Switzerland). After homogenizing the muscle tissue in PBS (1:10 *w*:*v*), the levels of blood urea nitrogen (BUN), lactic acid (LA), lactate dehydrogenase (LDH), creatine kinase (CK), and glycogen in the serum and tissue homogenate were assessed using kits (Nanjing Jiancheng Biotechnology Institute, Nangjing, China) with specifications. The levels of IL-6 and LPS in the serum were measured under the ELISA assay kit guideline (Fankew, Shanghai FANKEL Industrial Co., Ltd., Shanghai, China).

### 2.5. Quantitative Real-Time PCR (q Rt-PCR)

We followed the instructions provided by the kit (Vazyme Biotech Co., Ltd., Nanjing, China) to extract RNA from mouse gastrocnemius muscle tissues. The steps typically involve homogenizing the tissue, lysing cells to release RNA, and purifying the RNA using column-based or phenol–chloroform extraction methods. We ensured that the extracted RNA met the purity criteria, including having a concentration of ≥100 ng/μL, using a Nano-200 nucleic acid quantifier. After obtaining purified RNA, we proceeded to synthesize cDNA via a reverse transcription reaction using a suitable kit according to its instructions. We diluted the synthesized cDNA to an appropriate multiple for further analysis. For qPCR analysis, we performed the reaction according to our chosen qPCR kit’s instructions. We designed specific primers for each gene of interest based on their sequences, which were obtained from the NCBI database (Table 1). β-actin was used as the reference gene to normalize target gene expression levels. This reference gene was intended to exhibit stable expression across samples. We calculated the relative expression levels using the 2^−ΔΔCt^ method, which involved comparing Ct values (cycle threshold) between samples and controls after normalization with β-actin.

### 2.6. Western Blot Analysis

The muscle and colon tissue samples were lysed with RIPA lysis buffer (1:10 *w*:*v*), ground (60 Hz, 45 s, 3 times) and centrifuged (13,000× *g*, 20 min, 4 °C). The supernatant was collected as the sample protein, boiled with 25% loading buffer (5–10 min, 100 °C), and separated on SDS-PAGE. Proteins from the tissues were subjected to separation on 10% sodium dodecyl sulfate-polyacrylamide gels at 120 V for 40 min, and then transferred onto PVDF membranes (Bio-Rad Laboratories, Hercules, CA, USA) at 300 mA for 1.5 h. The membranes were blocked at room temperature for 15 min before being incubated with primary antibodies (refer to Table 2) overnight at 4 °C. Subsequently, the membranes were incubated with secondary antibodies for two hours at room temperature. Chemiluminescence was conducted using an ECL reagent (Beyotime Biotechnology, Shanghai, China), and imaged by a Gel Image System (Bio-Rad, Hercules, CA, USA).

### 2.7. Colon Histological Analysis

Fresh colon tissue was preserved in a 4% solution of paraformaldehyde for a minimum of 36 h, after which it was embedded in paraffin. The paraffin-embedded colon tissue was then sectioned into slices 5 μm in thickness and stained using hematoxylin and eosin. An inverted microscope was utilized to examine the pathological changes in the colon tissue.

### 2.8. 16S rDNA Gene Sequencing and Analysis

The genomic DNA was isolated from the fecal samples using a purification kit provided by Sangon Biotech Co., Ltd. (Shanghai, China). This DNA was then used to amplify the V3-V4 region of bacterial 16S rRNA genes, utilizing primer pairs 341F and 806R under specific PCR conditions. These included an initial denaturation at 95 °C for two minutes, 25 cycles of denaturation at 95 °C for thirty seconds, annealing at 55 °C for thirty seconds, and extension at 72 °C for thirty seconds, with a final extension step lasting five minutes performed at a temperature of 72 °C [25].

The amplified PCR products were then analyzed on two percent agarose gel via electrophoresis to verify their size and purity. The bands were recovered and purified with the AxyPrep DNA kit from Axygen Biosciences (Union City, CA, USA). The purified PCR products underwent quantitative detection using QuantiFluor™-ST from Promega (Madison, WI, USA), after which sequencing libraries were prepared for the samples that met the quality control standards. These libraries were sequenced on the NovaSeq platform. The raw sequence data were processed using the QIIME2 software (Version 1.9.1) suite, where paired-end sequences were merged after removing barcode/linker sequences.

For bacterial species identification based on their respective16S rRNA genes, the Greengenes database served as the reference database. Predictive functional profiling of microbial communities was performed using the Tax4Fun software package in R software (Version 4.3.1). The alpha diversity was analyzed using the “vegan” and “picante” packages in R software (Version 4.3.1). BugBase analysis was used to predict the biologically interpretable traits on the website (http://bugbase.cs.umn.edu (accessed on 17 October 2022)) [26].

### 2.9. Data Analysis

All experimental procedures were replicated at least three times, with results expressed as the mean ± standard deviation. Parametric data were analyzed using one-way ANOVA in GraphPad Prism 9.4.1, with significance determined at * *p* < 0.05, ** *p* < 0.01, *** *p* < 0.001, and **** *p* < 0.0001. In some tests, we identified extreme outliers due to experimental error or individual variation; these were excluded from the analysis, resulting in a smaller sample size. We ensured that all data were rigorously tested for statistical significance despite the smaller sample size.

The NMDS analysis was analyzed via the R package “vegan” and exhibited by the “ggplot2” package. Correlation analysis was conducted using the Pearson method and visualized via R package (“Pheatmap”). The gut microbiota data were processed with QIIME2, and visualizations were created using R Studio software (Version 4.3.1).

## 3. Results and Discussion

### 3.1. Nutrient Intake of Mice

#### Measurement of AEB Main Components

As this experiment used the same raw materials as those previously examined, we can assume that the proportion of micromolecules was similar [27]. The primary components of the AEB lyophilized powder were measured and are presented in Table 3. The energy content was calculated through this formula: Total Energy (kcal) = (grams of carbohydrates × 4) + (grams of proteins × 4) + (grams of fats × 9). The AEB oral supplement provided each mouse with roughly 0.821 cal/g/day (low dose) or 1.642 cal/g/day (high dose) relative to their body weight.

### 3.2. Anti-Fatigue Effect of Brassica rapa L. (AEB)

#### 3.2.1. Effect on Fatigue-Related Biomarkers

**Figure 2:**
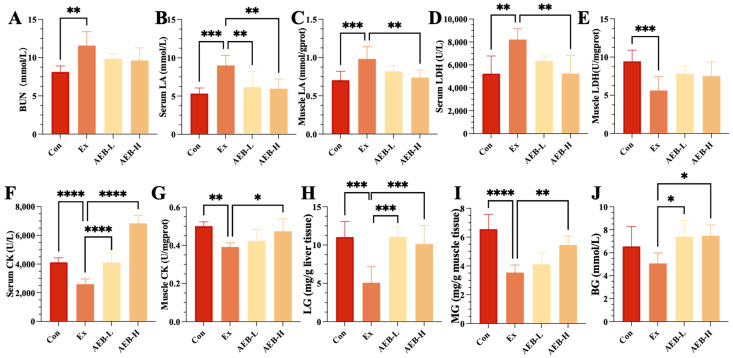
Effects on fatigue-related biomarkers in mice. (**A**) BUN, (**B**) BLA, (**C**) MLA, (**D**) serum LDH, (**E**) muscle LDH, (**F**) serum CK, (**G**) muscle CK, (**H**) LG, (**I**) MG, (**J**) BG. * *p* < 0.05, ** *p* < 0.01, *** *p* < 0.001, and **** *p* < 0.0001. (*n* = 8).

To assess the anti-fatigue effect of AEB, we measured typical peripheral fatigue-related biomarkers [28]. These results are summarized in Figure 2. The BUN level in the Ex group was 42.39% higher than that of the Con group (*p* < 0.01). Although AEB treatment showed a trend towards reducing BUN levels, neither the low-dose AEB (AEB-L) nor the high-dose AEB (AEB-H) groups achieved significant efficiency. In serum, LA levels were also significantly elevated in the Ex group, being 69.05% higher than in the Con group (*p* < 0.001). Both doses of AEB treatment exhibited a trend towards decreasing LA levels in serum (*p* < 0.01), and this trend was mirrored in the muscle tissue. LDH levels in serum increased by 56.71% in the Ex group compared to the Con group (*p* < 0.01), with the lowest LDH level observed in the AEB-H group. Interestingly, while LDH levels decreased by 40.45% in muscle tissue after exercise, they increased following both doses of AEB treatment. CK levels decreased significantly post-exercise within serum samples (*p* < 0.001), but were enhanced following intervention with either dose of AEB—a pattern that was similarly observed within muscle tissue.

Overall, these findings suggest that AEB treatment can reduce protein metabolism during exercise, as shown by the reduced BUN levels; improve aerobic metabolism by decreasing lactic acid; and decrease muscle damage, as indicated by the lowered LDH and CK levels. These results support the potential anti-fatigue effects of AEB in our current research.

#### 3.2.2. Effect on Glycogen Storage

The quantification of glycogen levels in the muscle and liver serves as a direct reflection of the body’s energy storage status. These measurements offer a straightforward insight into the dynamics of energy metabolism within the system. As shown in Figure 2, it was found that the contents of liver glycogen (LG) and muscle glycogen (MG) in the Ex group were lower than those in the Con group by 53.97% (*p* < 0.001) and 45.91% (*p* < 0.001). Meanwhile, AEB treatment led to an increase in the contents of LG and MG. Notably, the high-dose AEB group exhibited significant increases of 99.36% (*p* < 0.05) and 53.90% (*p* < 0.01), respectively, compared to the Ex group.

The storage of glycogen plays a pivotal role in ensuring the stability of blood glucose (BG) levels and facilitating the synthesis of adenosine triphosphate (ATP) during exercise [27]. As depicted in Figure 2J, BG levels within the Ex group exhibited a marginal decrease compared to those in the Con group, although this difference lacked statistical significance. Remarkably, AEB treatment resulted in a noteworthy elevation of blood glucose levels by 47.32% when contrasted with the Ex group (*p* < 0.05).

These experimental results indicate that AEB intake promotes oxidative phosphorylation by increasing glycogen storage, thereby stabilizing energy metabolism and exerting an anti-fatigue effect. Given these changes in glycogen storage levels, we next investigated the impact of AEB on the energy metabolism function within muscle tissue.

### 3.3. Effect of AEB on Energy Metabolism Function in Muscle

#### 3.3.1. Effect on Glycolysis Process

Given the critical role of the TCA cycle in energy production through glucose metabolism, we examined the gene expression of several related enzymes to assess the homeostasis of glycolysis in muscle tissue.

**Figure 3:**
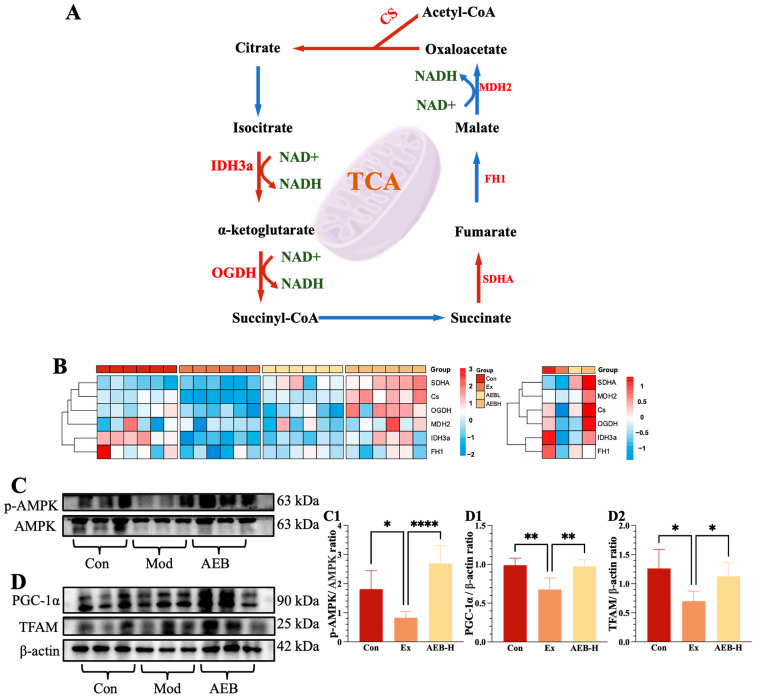
Effects on TCA-cycle-related genes. (**A**) Genes acting in the TCA cycle, (**B**) relative expression of genes. (*n* = 8). (**C**,**D**) p-AMPK, AMPK, PGC-1α, and TFAM protein blot bands. (**C1**,**D1**,**D2**) Relative expression of p-AMPK, PGC-1α, and TFAM. * *p*< 0.05, ** *p* < 0.01 and **** *p* < 0.0001. (*n* = 6).

In this study, the analyzed genes included the citrate synthase (CS), isocitrate dehydrogenase 3a subunit (IDH3a), α-ketoglutarate dehydrogenase (OGDH), succinate dehydrogenase complex flavoprotein subunit A (SDHA), fumarate hydratase 1 (FH1), and malate dehydrogenase 2 (MDH2) [29]. As seen in Figure 3A,B, it is evident that chronic fatigue significantly impacted the expression of CS and IDH3a (*p* < 0.001). However, intervention with AEB appears to have mitigated this disruption by maintaining IDH3a and OGDH at their normal levels and significantly augmenting the expression of the CS and SDHA genes. In contrast, the expression levels of FH1 and MDH2 remained stable throughout this experiment.

These results suggest that AEB treatment significantly influenced the expression of crucial enzymes involved in the TCA cycle. This intervention appeared to stimulate the initial catalytic steps of the TCA cycle within muscle tissue, enhance intermediate processes for increased ATP production, and sustain terminal processes to ensure continuity into subsequent cycles. As the mitochondria are the primary site of the TCA cycle [30] we subsequently investigated the mitochondrial function in muscle tissue. Furthermore, some current research has illustrated that glycolysis plays an important role in the inflammatory response; thus, we studied this afterward [31].

#### 3.3.2. Effect on Mitochondrial Function of Muscle

The role of mitochondria is important in energy generation, but the inflammatory response can also be reflected. The results shown in Figure 2 and Figure 3A indicate that the high dosage had the most significant impact, while the effects at lower dosages were relatively minor. It is believed that these results in the high-dosage group best illustrate the potential impact of *Brassica rapa* L. administration.

As depicted in Figure 3C,D, the phosphorylated AMP-activated protein kinase (p-AMPK) expression in the Ex group exhibited a reduction of 54.33% (*p* < 0.05), while the administration of AEB led to a substantial increase of 225.03% (*p* < 0.001). Additionally, a significant elevation in the expression of peroxisome proliferator-activated receptor gamma coactivator-1 alpha (PGC-1α) was observed with AEB treatment in comparison to the Ex group (*p* < 0.01). Furthermore, the relative expression of mitochondrial transcription factor A (TFAM) was notably higher in the AEB group, by 87.72% (*p* < 0.01), as compared to the Ex group. Collectively, these results highlight that AEB treatment can potentially enhance mitochondrial biogenesis through the upregulation of the AMPK/PGC-1α/TFAM signaling pathway. This modulation contributes to the maintenance of the respiratory chain’s equilibrium within muscle cells.

### 3.4. Effect of AEB on Inflammatory Response

#### 3.4.1. Effect on TLR4/NF-κB Pathways

Numerous studies have corroborated the complex connection between mitochondrial function and inflammatory responses [32]. Any disruptions in mitochondrial function can act as potent instigators, triggering inflammatory responses. It has also been established through research that prolonged and high-intensity physical activity can frequently lead to the activation of NF-κB pathways and contribute to chronic inflammation [33], with the muscle and intestinal ecosystem experiencing the most impact [34]. In these inflammatory processes, invading pathogens or stress reactions are invariably present, with Toll-like receptor 4 (TLR4) as a key agent. TLR4 is essential for recognizing these elements, which sets off a sequence of reactions that lead to the activation of subsequent NF-κB-related inflammatory signaling pathways [35].

Within the scope of this study, our observations unveiled significant elevations in the expression levels of TLR4, NF-κB, and IL-6 within the Ex group when contrasted with the Con group (*p* < 0.01). However, the administration of AEB, particularly in the AEB-H group, yielded conspicuous reductions in the expression levels of these pivotal inflammatory markers in comparison to the Ex group. As verified by ELISA measurement of IL-6 levels in serum, it can also be observed that the Ex group experienced a significant increase compared with the Con group (*p* < 0.001), while high-dose AEB treatment notably reduced these levels (*p* < 0.05). Thus, the inflammatory statues were initially estimated by identifying the NF-κB pathways activated by TLR4.

#### 3.4.2. Effect on LPS Levels

**Figure 4:**
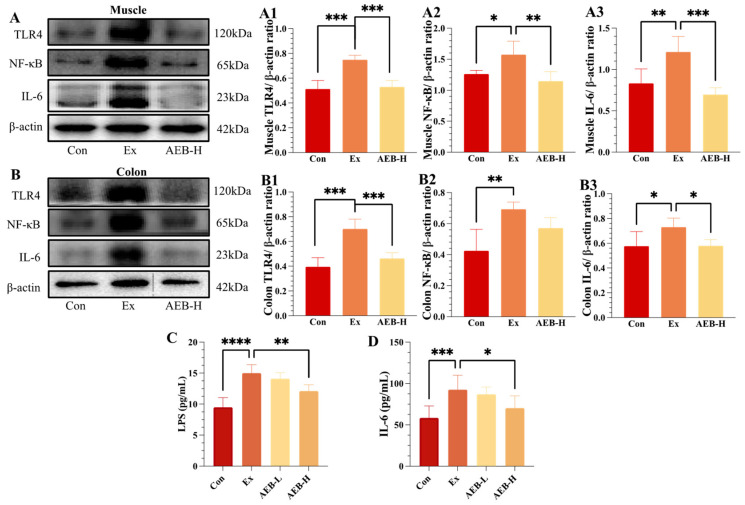
Effects on the expression of inflammatory responses. (**A**) TLR4, NF-κB, and IL-6 protein blot bands in muscle. (**A1**–**A3**) Relative expression of TLR4, NF-κB, and IL-6 in muscle. (**B**) TLR4, NF-κB, IL-6 protein blot bands in colon. (**B1**–**B3**) Relative expression of TLR4, NF-κB, and IL-6 in colon (*n* = 3). (**C**) LPS levels in serum (*n* = 8). (**D**) IL-6 levels in serum. * *p* < 0.05, ** *p* < 0.01, *** *p* < 0.001, and **** *p* < 0.0001. *(n* = 8).

The recognition of LPS infection triggers the activation of the Toll-like receptor (TLR), subsequently initiating inflammatory pathways [36]. In this study, we observed activation within the TLR4 pathway, indicating a potential cause of inflammation in both the muscle and the colon. As depicted in Figure 4C, intensive exercise led to a significant increase of 58.05% (*p* < 0.001) in LPS levels in the Ex group compared to the Con group. However, AEB treatment displayed a remarkable ability to effectively counteract this elevation in LPS levels. Particularly, the AEB-H group exhibited a notable reduction of 19.29% (*p* < 0.01) in LPS levels compared to the Ex group.

These results imply that AEB could potentially harbor anti-inflammatory properties, offering the potential to mitigate exercise-induced inflammation and muscle injury by modulating LPS levels and regulating the TLR4/NF-κB signaling pathway. These findings warrant further investigation into the origins of LPS infection, which may potentially be associated with intestinal microbiota [37].

### 3.5. Effect of AEB on Intestinal Integrity

Intestinal integrity has emerged as a pivotal factor within the intricate interplay of the gut–muscle axis, profoundly influencing communication between the gut and other organs, including skeletal muscles. The robustness of the intestinal barrier assumes a crucial role in upholding a delicate equilibrium of nutrients, immune cells, and signaling molecules. Additionally, it acts as an effective barrier, thwarting the translocation of detrimental substances like endotoxins and pathogenic bacteria. Moreover, the intestinal barrier assumes a virtual sentinel role in preventing LPS infections triggered by enterogenous Gram-negative bacteria [38].

**Figure 5:**
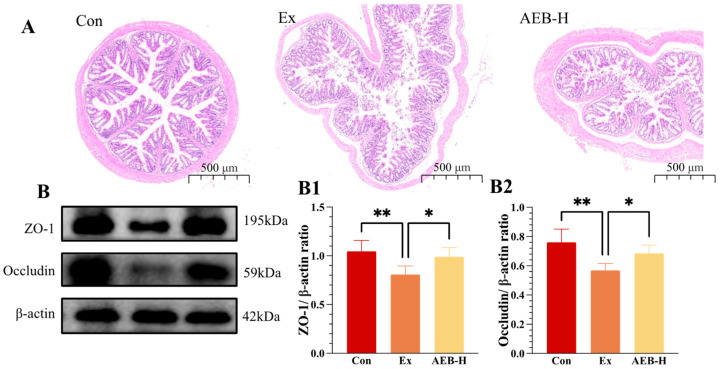
Effects on intestinal integrity. (**A**) H&E staining of the colon; (**B**) ZO-1, occludin protein blot bands. (**B1**,**B2**) Relative expression of ZO-1 and occludin. * *p* < 0.05, ** *p* < 0.01. (*n* = 3).

As depicted in Figure 5A, the colonic epithelial cell structure in the Con group exhibited notable alignment, while the crypt structure remained within normal parameters. Subsequently, the exercise-induced group exhibited a degree of colonic villi damage. However, in comparison to the Ex group, the AEBs group demonstrated comparatively milder colonic lesions, with a more intact crypt structure.

The modulation of intestinal barrier function is underpinned by tight junctions, intricate complexes connecting adjacent intestinal epithelial cells. Key components include occludin, an integral plasma membrane protein, and ZO-1, an outer membrane protein. As illustrated in Figure 5B, the Ex group displayed a reduction of 25.27% in ZO-1 expression and 22.82% in occludin expression compared to the Con group. Notably, AEB intervention remarkably counteracted these effects, inducing significant reversals of 19.13% in ZO-1 levels and 21.44% in occludin levels.

These findings underscore the susceptibility of the intestinal barrier to disruption due to rigorous exercise, resulting in compromised integrity. Such disturbances render the body more susceptible to conditions such as LPS infection, subsequently triggering a cascade of reactions, including inflammation. Notably, AEB is presumed to exert its previously observed functional effects by initially safeguarding intestinal integrity.

### 3.6. Effect of AEB on Gut Microbiome

#### 3.6.1. Changes in Microbial Diversity

To substantiate the impact of enterogenous bacteria on these biological processes, we employed 16s rDNA analysis to delve into the intricacies of gut ecology.

**Figure 6:**
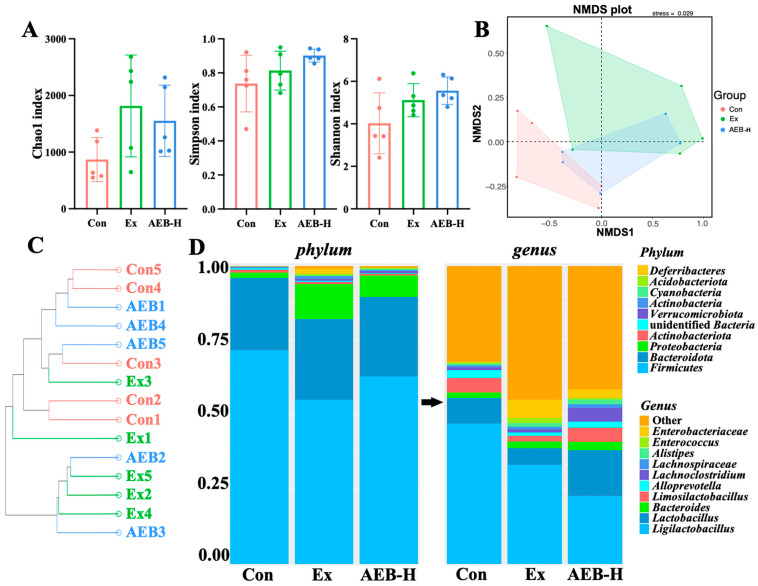
Effects on microbial diversity. (**A**) Alpha diversity. (**B**) NMDS diversity. (**C**) Clustering tree diagram. (**D**) Alterations in microbiota at the phylum and genus levels. (*n* = 5).

The Chao1 index represented the total number of species. The Simpson and Shannon indices represented microbial diversity [39]. As depicted in Figure 6A, in comparison to the mice in the Con group, the consequences of excessive exercise-induced fatigue were evident in the observed increase in the Chao1 index. Strikingly, the AEB group showcased a divergent trend where the Chao1 index demonstrated a decrease, implying that AEB holds the potential to counteract the exercise-induced surge in microbial abundance. The Simpson and Shannon indices echoed a similar narrative, revealing that exercise amplified the diversity and uniformity of the microbiota when compared to the Con group. Intriguingly, the AEB treatment further heightened these indices compared to the Ex group. This collective insight underscored an overarching enhancement in the richness and evenness of the gut microbial community due to exercise and further augmentation through AEB intervention.

The NMDS analysis provided a visual representation of the inter- and intra- group variance across the samples. Illustrated in Figure 6B, the Ex group stood distinctly apart from the Con group, signifying that chronic exercise intervention yielded significant alterations in the composition of the intestinal microbial community. In contrast, the AEB group occupied an intermediary position between the Ex and Con groups, highlighting the regulatory role of AEB in fatigue modulation. This regulation was underscored by its capacity to modulate the composition and abundance of gut microbes across the groups, contributing to a comprehensive understanding of fatigue regulation.

*Firmicutes*, *Bacteroidota*, and *Proteobacteria* were the dominant phyla. As shown in Figure 6C, the relative proportions of these phyla changed significantly. The dysbiosis in the gut microbiota is commonly associated with *Proteobacteria*. Fatigue-induced increases in phylum *Proteobacteria* have also been linked to intestinal inflammation and oxidative stress. Furthermore, comparing the Ex group with the Con group, the proportions of *Firmicutes* decreased, and the F/B (*Firmicutes*/*Bacteroidota*) ratio increased, while the proportion of *Proteobacteria* increased. When it comes to AEB group, the composition of gut microbes was closer to Con group, implicating that AEB reversed the fatigue-related changes in the composition of the F/B ratio.

#### 3.6.2. The Phenotypes of Microbial

**Figure 7:**
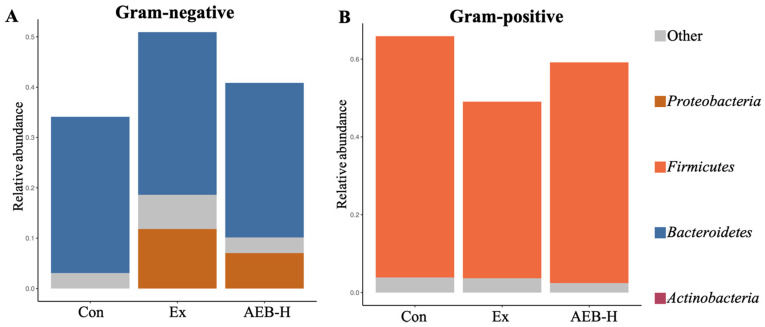
Phenotypic distribution. (**A**) Gram-negative bacteria. (**B**) Gram-positive bacteria. (*n* = 5).

The phenotypes of microbiomes always reflect their potential effects [26]. According to the prediction of Gram phenotypes, Gram-negative bacteria are mainly composed of *Bacteroides* and *Proteobacteria*. Seen in Figure 7, the abundance of Gram-negative bacteria in Ex group was 49.33% higher than that in the Con group, and that in the AEB group was 19.81% lower than that in Ex group. Gram-positive bacteria are mainly composed of *Firmicutes*. The abundance of Gram-positive bacteria in the Ex group was 25.54% lower than that in the Con group, and that in the AEB group was 20.57% lower than that in the Ex group.

#### 3.6.3. Correlation Analysis of Gut Microbiome and Biochemical Parameters

**Figure 8:**
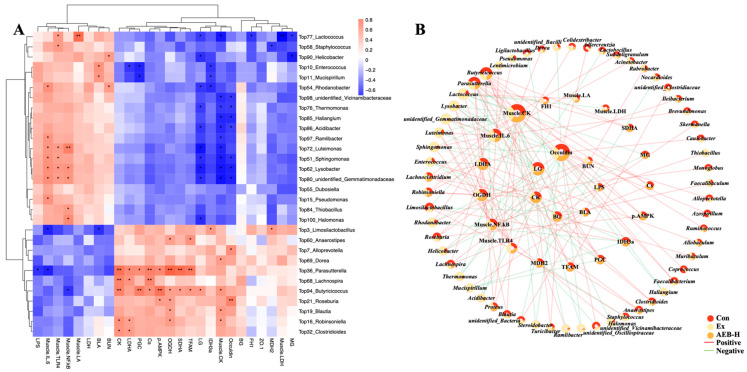
Microbial correlation with biochemical parameters. (**A**) Correlation analysis of the gut microbiome and biochemical parameters. (**B**) Network image of correlation. * *p* < 0.05, ** *p* < 0.01. (*n* = 5).

Pearson correlation analysis was employed to assess the inherent relationship between the gut microbiome (on genus levels) and various biochemical parameters, aiming to unveil the connection between gut microbiome and muscle function. The results of this analysis are visually depicted in Figure 8A as a heatmap. In this heatmap, markers that exhibited heightened levels due to prolonged exercise, such as BUN, LA, LPS, and IL-6, whose elevation was associated with unfavorable physiological responses, are clustered together. Conversely, beneficial indicators that can be increased through AEB treatment, such as occludin protein levels, glycogen storage markers, and factors associated with energy metabolism, are also grouped separately. Consequently, microbiomes exhibiting positive correlations with these disorder indices and negative correlations with those beneficial indicators can be identified as pernicious bacteria; conversely, they can be considered to be probiotics. We discovered that *Butyricicoccus*, *Parasutterella, Roseburia,* and *Lachnospira* exerted the most positive influence in this experiment. On the other hand, *Lactococcus*, unidentified_*Gemmatimonadaceae*, *Lysobacter,* and *Luteimonas* demonstrated the most negative effects.

We filtered the correlation results to include only data with |R| ≥ 0.5 and |*p*| ≤ 0.05. The filtered data were then used to establish the correlation network. In the network diagram (Figure 8B), the correlation and proportional distribution among different groups of materials are clearly depicted.

The microbiomes are represented in the outer ring, while the muscle-related indices are displayed in the middle ring, and other indices from serum and other tissues are shown in the central ring. This visualization greatly facilitates the recognition of how AEB treatment transformed these microbial communities. It is evident from the diagram that AEB treatment leads to a significant reduction in harmful bacteria such as *Enterococcus*, *Sphingomonas*, *Mucispirillum*, *Pseudomonas*, and others.

## 4. Discussion

Building on our previous work, which revealed the anti-fatigue potential of *Brassica rapa* L. roots in acute stress models, we extended our investigation to a chronic model [40,41]. This chronic model reflects the persistent nature of stress experienced by many people today. Our study used prolonged and intense forced swimming as a chronic model to elicit the enduring effects on both peripheral and inherent components. This experiment aimed to scrutinize the broader changes induced by long-term stress—specifically alterations in fatigue-related biomarkers, energy metabolism genes, inflammatory responses, intestinal integrity, and gut microbiota composition. Through this comprehensive approach examining the bioactive impact of AEB intervention under chronic stress conditions, we aimed to further elucidate the mechanisms underlying *Brassica rapa* L.’s beneficial effects.

Firstly, we tested how well *Brassica rapa* L. fights fatigue by measuring common fatigue markers, like BUN, LDH, LD, and CK levels in the blood and muscle. The fluctuation of BUN levels provides a clear indication of heightened protein catabolism to fuel the demands of intense exercise. While the oral supplementation materials exhibited a limited capacity to revert this change, it is noteworthy that the significant reduction in lactic acid accumulation underlined AEB’s potential to exert substantial control over the progress of anaerobic glycolysis. In fact, reports have proven that *Brassica rapa* L. can enhance the activity of erythrocyte and the level of mean corpuscular hemoglobin concentration in the body, thus transporting more oxygen [42]. By introducing AEB, this optimization provides a greater opportunity for cells to engage in aerobic metabolism, potentially offsetting the challenges of extreme exercise-induced anaerobiosis. The differences observed in LDH levels between the serum and muscles can be attributed to the physiological characteristics of LDH and the dynamics of its release and clearance in the body. LDH is an enzyme that is present in various tissues, including muscle. When muscle cells are damaged or injured, LDH is released into the bloodstream. This leads to an increase in LDH levels in the serum [43]. As the LDH level tendency seems to be similar to our previous study, the CK levels were quite different [27]. We observed an elevation of CK levels in serum coupled with a decline in its concentration within muscle tissue following instances of acute exercise [27]. However, with the long-term exercise treatment in this experiment, the concentrations in both the serum and muscle tissue were decreased. Although strenuous exercise might inflict damage upon muscle cells, prompting the release of CK stored within these cells into the bloodstream, clinical observations have revealed an interesting contrast: patients afflicted by chronic fatigue consistently exhibit low CK levels [44]. The diminished synthesis of CK ultimately disrupts ATP synthesis. Given this understanding, we can assume that the mice subjected to this experiment may have experienced energy insufficiency, which prompts us to delve more precisely into the intricacies of muscle energy metabolism function [45].

Our data uncovered that following the experimental regimen, the expression of genes associated with the glycolysis process exhibited a tendency towards disorder, with notable impacts observed in CS, IDH3a, and FH1. However, upon the administration of AEB supplementation, these expressions returned to normal levels. This suggests that the effects of AEB might be associated with the restoration of glycolytic balance. As the primary sites of glycolysis, mitochondria play a pivotal role in this process. Intensive exercise caused the expression of the AMPK/PGC-1α/TFAM signaling pathway to decrease, indicating the mitochondrial biogenesis might have been disturbed, and the implementation of AEB modulated it. This modulation contributed to the maintenance of the respiratory chain’s equilibrium within muscle cells, revealing that mitochondria could potentially be one of the underlying targets of AEB’s action.

The mitochondria can also play an intervening role in the toll-like receptor-mediated innate immune responses and inflammasome complex activation [32]. Within this interplay, TLR4 emerges as a pivotal player, orchestrating the recognition of invading pathogens and the activation of NF-κB inflammatory cascade [46]. The outcomes of our study have unveiled inflammation responses occurring in both the muscle and colon that are triggered by intense exercise. Notably, intervention with AEB exerted substantial mitigating effects on the expression levels of these pivotal inflammatory markers. This reduction underscores AEB’s potential as an anti-inflammatory agent, with the capability to counteract the heightened inflammation typically observed during exercise. As reported, LPS is recognized as a trigger for Toll-like receptor activation [47]. Our outcome found the tendency of LPS to fluctuate is correlated with the TLR4 expression between groups. This suggests that the inflammatory response in our experiment might be attributed to LPS infection. In fact, LPS is a component of the outer membrane of Gram-negative bacteria [36]. Therefore, it can be hypothesized that the process of inflammatory response initiates from the gut. Additionally, various studies conducted on individuals with chronic fatigue syndrome (CFS) have identified compromised intestinal barrier integrity [36,37]. This body of evidence bolsters our hypothesis regarding the origin of inflammation and aligns with our emphasis on the significance of intestinal integrity.

Upon conducting morphological observations and assessing tight junction protein levels in colon tissue, a clear pattern emerged: prolonged exercise leads to evident impairment of intestinal barrier integrity, and the administration of AEB had the potential to counteract this impairment, effectively restoring the integrity of the intestinal barrier. This observed alteration has provided compelling evidence that intense exercise has the capacity to elevate the presence of Gram-negative bacteria, consequently undermining the integrity of the intestinal barrier. As these Gram-negative bacteria breach the permeable gut barrier into the bloodstream, they trigger the activation of TLR4 in other tissues (muscle), thereby initiating an inflammatory signaling cascade involving the activation of NF-κB/IL-6 pathways. However, the pathway of LPS to reach the muscle tissues is indeed complex, and further research should be implemented to investigate the dynamics of the LPS infection process. As recent research has pointed out, LPS exposure can activate the K+ current and cause hyperpolarization in the muscle tissue of larval *Drosophila* [47]. This theoretical construct accentuates the evolving interdependence between the gut and muscle within our research, illustrating the emergence of an embryonic-formed gut–muscle axis.

Due to the vitality Gram-negative bacteria levels in this gut–muscle axis hypothesis, 16S rDNA analysis was utilized to confirm the distribution of the intestinal microbiome. Our results proved that an intensive exercise regimen significantly alters the composition of the microbiome. The administration of AEB treatment successfully preserves this composition, distinguishing itself from both the Con and the Ex groups. After the phenotypes were obtained by microbial analysis, we finally identified the increment in average abundance of Gram-negative bacteria after following the exercise schedule. AEB intervention effectively counteracted this aberrant trend, particularly manifesting in a reduction in the ratio of *Proteobacteria* to *Bacteroidetes*.

Last but not least, in order to further investigate the underlying mechanisms by which the microbiome influences peripheral reactions and contributes to the modulation of fatigue through the gut–muscle axis, correlation analysis was conducted between the intestinal microbiome and various biochemical parameters. Our findings revealed that AEB can inhibit several pernicious bacteria, such as *Enterococcus*, *Sphingomonas*, *Mucispirillum*, and *Pseudomonas*, and their roles are as follows:

(1) *Enterococcus* leads to serious infection in the body, and inducing the inflammatory response. As previously reported, an increase in Enterococcus abundance has been found in patients with Parkinson’s disease, suggesting a possible link to neuroinflammation [48]. Furthermore, another study found that inhibiting *Enterococcus* could alleviate hepatic inflammation [49]. *Enterococcus*, among the top ten most abundant genera in the microbiome, significantly correlated with increased lactic acid during exercise and inhibited PGC-1α expression, potentially disrupting muscle energy metabolism. Furthermore, it positively correlated with inflammatory markers. These suggests that *Enterococcus* can impact exercise physiology and inflammation.
(2) As reported, *Sphingomonas* was enriched in the feces of PD patients, suggesting a potential role in PD pathogenesis or response [50]. This bacterium can cause infections in immunocompromised individuals. In this research, it showed significant correlation with inflammatory responses and was associated with a strain on intestinal integrity. These findings suggest that *Sphingomonas* may contribute to inflammation and disrupt gut health.
(3) Alterations in *Mucispirillum’s* abundance and function have been observed in certain disease conditions, such as inflammatory bowel disease and obesity [51]. In this research, it showed a positive correlation with the inflammation indices and was associated with mitochondrial disorders.
(4) *Pseudomonas*, a known pathogenic bacterium, is often associated with certain neural diseases and is considered a trigger for neural inflammation [52]. In our research, we also observed its significant correlation with inflammation.

Furthermore, other bacteria, such as *Luteimonas*, *Lysobacter*, and *Lactococcus*, may also be suspected to induce various harmful effects in this experiment. Whether their mechanisms of action are similar or dissimilar to those mentioned above, their physiological impacts via gut–muscle axis were observed in this study.

The probiotic bacteria, which demonstrated a positive correlation with modulation pathways and negative correlation with impairment pathways in this study, include the following:

(1) *Parasutterella* can link fatty acid biosynthesis pathways, enhancing the energy metabolizing progress and reducing the risk of obesity [53]. In this study, *Parasutterella* showed a significant correlation with these energy metabolism pathways. Additionally, it was found to inhibit the progression of infections towards inflammation.
(2) *Butyricicoccus* is named due to its ability to produce butyrate. Butyrate can serve as an energy source for colonocytes and help to maintain the integrity of the intestinal barrier. It has anti-inflammatory properties [54], and was also found to inhibit NF-κB activity [55].
(3) *Roseburia* is also a bacterium that can induce the production of butyrate [56].
(4) *Lachnospira* can help to metabolize dietary carbohydrates and produce SCFAs such as acetate and butyrate [57].

In conclusion, these probiotic bacteria can act as inflammation inhibitors to mitigate the damage caused by prolonged exercise stress. Additionally, research into these bacteria has directed their function in terms of producing SCFAs, particularly butyrate. This has piqued our interest in further investigating the metabolic pathways of the intestinal microbiome, with an aim to discover more targets that can be influenced by gut activity induced by dietary supplements of AEB. This could establish more host–microbiome connections, such as the gut–liver or gut–brain axes.

One potential limitation of our study is that some analyses included fewer than eight mice due to outliers and resource limitations. While we took measures to ensure the robustness of our results through replication and rigorous statistical testing, this smaller sample size may have influenced certain findings. Future studies with larger sample sizes are needed in order to confirm these preliminary findings and to further investigate the effects of *Brassica rapa* L. on fatigue during prolonged exercise.

## 5. Conclusions

Our study elucidated the multifaceted role of *Brassica rapa* L. in modulating energy metabolism, controlling inflammation, and preserving intestinal integrity under conditions of chronic exercise-induced stress. We found that AEB supplementation effectively restored the glycolytic balance and mitigated mitochondrial disturbances induced by intensive exercise while reducing inflammation in muscle and colon tissues. Furthermore, it counteracted the exercise-induced increase in harmful Gram-negative bacteria and maintained intestinal barrier integrity. Correlation analysis identified several pathogenic bacteria linked to fatigue through their effects on inflammation and energy metabolism, as well as probiotic bacteria beneficial for energy metabolism enhancement and inflammation reduction. Ultimately, our research underscores the potential of *Brassica rapa* L., particularly its impact on gut microbiota, to manage chronic stress-induced fatigue, paving the way for future therapeutic applications.

## Figures and Tables

**Table 1:** Primer design.

Gene	Sense Primer 5′-3′	Anti-Sense Primer 5′-3′
CS	CAGAATTAAGACCTTCAAGCAGCAACA	TCAAGAACCGAAGTCTCATACACAAGT
IDH3a	GCAATGTCACAGCAATTCAAGGA	ACGAAGCAACAGATTCATAGATGGA
OGDH	GGAGGAGGAGGTGGCTATTAC	CCTTGGTTCTTGTGCTCTTCC
SDHA	GTGGACATCAAGACTGGCAAGGT	GTAGGAGCGGATAGCAGGAGGTA
FH1	TGTTACCGTTGGAGGCAGCAATG	GTCTGTGAAGGACACTGAAGCATCTC
MDH2	GGAAGGAAGGAGTCGTTGAGTGTT	ATCTTGCCAATGCCCAGGTTCTT

**Table 2:** Primary anti-bodies.

Primary Antibodies	Dilution Ratio	Manufacturer
p-AMPK	1:1000	Abcam, Cambridge, UK
AMPK	1:1000	Abcam, Cambridge, UK
PGC-1α	1:5000	Abcam, Cambridge, UK
TFAM	1:1000	Beyotime, Shanghai, China
TLR4	1:1000	Proteintech, Chicago, USA
NF-κB	1:2000	Cell Signaling Technology, Beverly, USA
IL-6	1:1000	Abcam, Cambridge, UK
ZO-1	1:2000	Abcam, Cambridge, UK
Occludin	1:2000	Proteintech, Chicago, USA
β-actin	1:1000	Proteintech, Chicago, USA

**Table 3:** Main components of AEB.

Main Components	Dehydrated *Brassica rapa* L. (g/100 g)	AEB (g/100 g)
Soluble sugar	12.364 ± 1.233	27.754 ± 1.764
Total protein	7.098 ± 0.616	12.764 ± 1.426
Total lipid	0.135 ± 0.007	0.234 ± 0.002
Total amino acid	4.171 ± 0.056	7.983 ± 0.168
Total Fiber	10.345 ± 0.732	2.432 ± 0.543
Total flavonoids	0.460 ± 0.089	1.343 ± 0.324
Total polyphenols	0.304 ± 0.071	2.533 ± 0.134
Total triterpenes	0.386 ± 0.059	3.412 ± 0.243
Total saponin	0.396 ± 0.025	2.754 ± 0.342

## Data Availability

All sequence data in this study are available in the National Center of Biotechnology Information (https://www.ncbi.nlm.nih.gov/bioproject/, accessed on 12 April 2022).

## Supplementary Materials

The following supporting information can be downloaded at: https://www.mdpi.com/article/10.3390/nu15224737/s1, Figure S1: Raw blot images for AMPK protein; Figure S2: Raw blot images for PGC-1α, TFAM protein; Figure S3: Raw blot images for TLR4, NF-κB, IL-6 protein; Figure S4: Raw blot images for ZO-1, Occludin protein. Table S1: Ingredient composition of animal feed; Table S2: Macronutrients in animal feed; Table S3: Micronutrients in animal feed.
