# Supplementary material for: Aqueous Extract of Brassica rapa L.’s Impact on Modulating Exercise-Induced Fatigue via Gut–Muscle Axis
Source: Nutrients. 2023 Nov 9;15(22):4737. doi: 10.3390/nu15224737 (PMC10674577; doi:10.3390/nu15224737)
Supplement: Supplementary file 1 [file nutrients-15-04737-s001.zip › nutrients-2695282-supplementary.pdf]

Underlying raw images of Western blot

In Figure S1, to ensure that our strip was cut from the same membrane, we marked it by cutting a corner. The antibody of the phosphorylated protein was initially incubated. After blotting, it was removed using a stripping buffer, and then the target protein's antibody was incubated. Which exhibited in the article is marked with a “Star”.

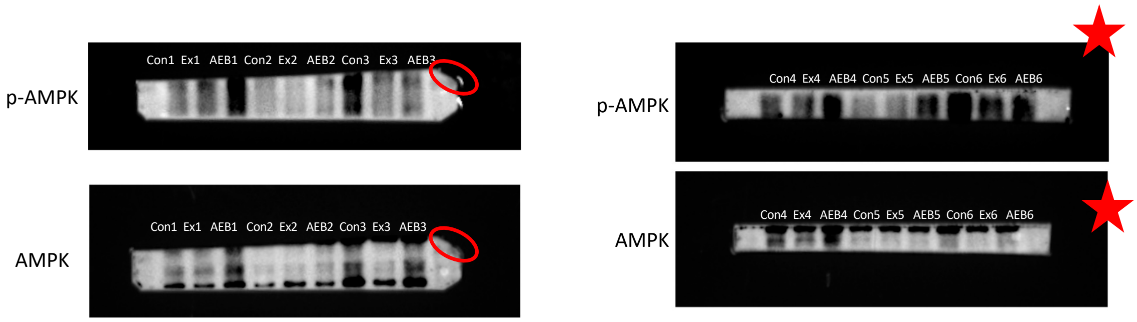

Figure S1 Raw blot images for AMPK protein

In Figure S2, to ensure that our strip was cut from the same membrane, we marked it by cutting a corner. The second membrane was incubated simultaneously with the first one. Therefore, the complete membrane is a cut from another identical membrane. Which exhibited in the article is marked with a “Star”.

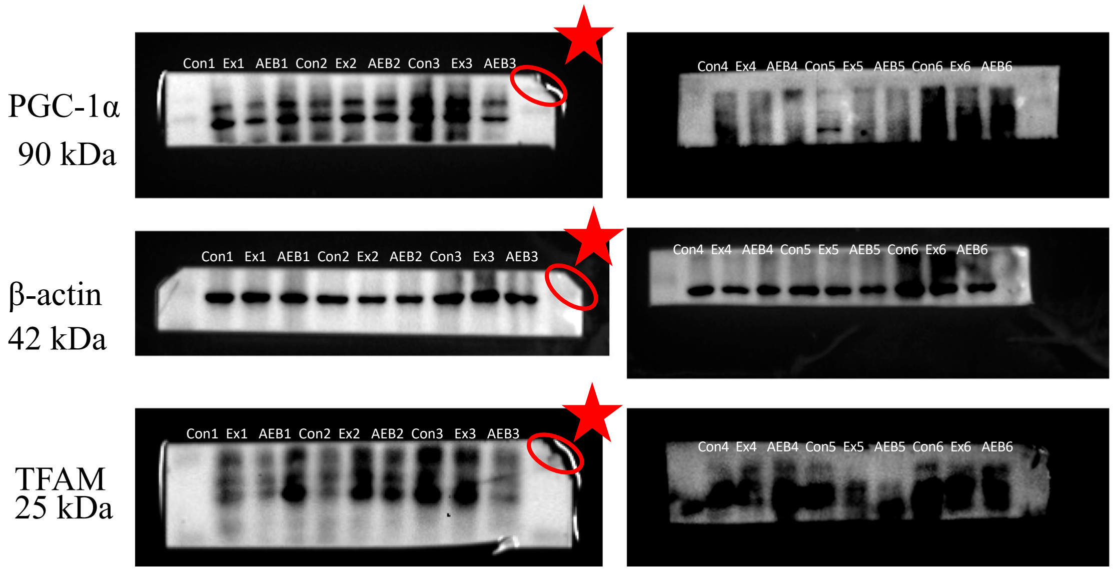

Figure S2. Raw blot images for PGC-1α, TFAM protein

In Figure S3, the blotting of muscle protein was not performed simultaneously with the colon protein. Although we neglected to clear the background, our image analysis has not been affected since the blot in the strip is explicit. Incidentally, because it was performed separately, the lanes marked as 'Sample' also represent our experimental samples. However, considering these are irrelevant to Brassica rapa L., we ultimately removed them. The portion exhibited in the article is highlighted within a red rectangle. And the strip in TLR4 of colon were straighten by ImageJ software.

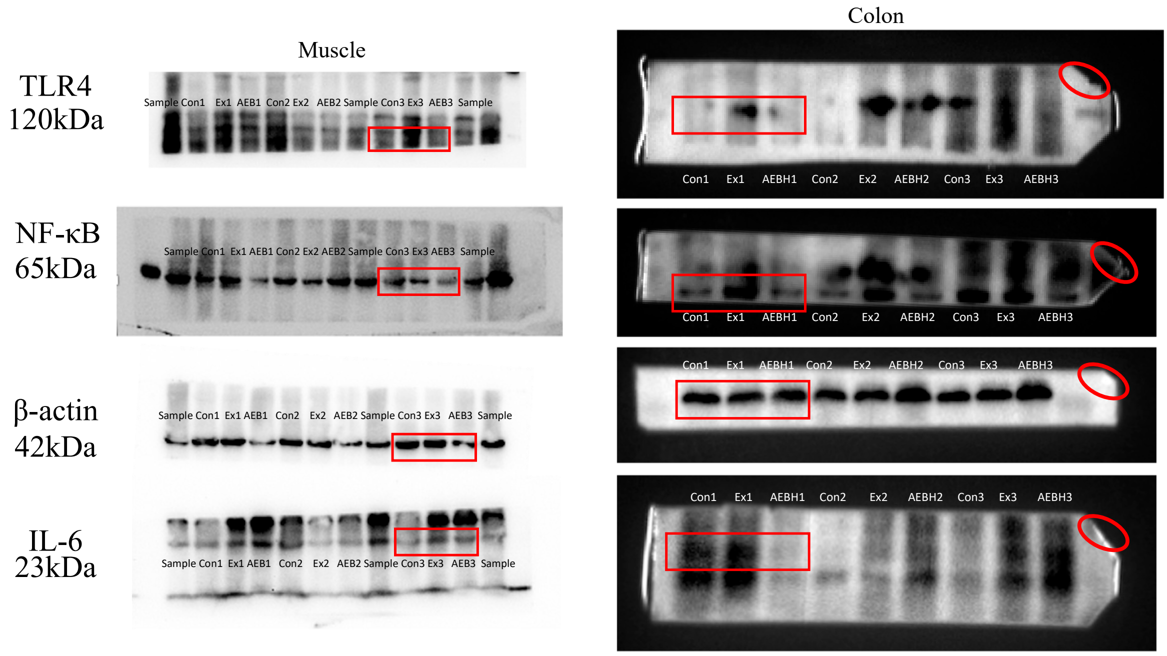

Figure S3. Raw blot images for TLR4, NF-κB, IL-6 protein

In Figure S4, we cut the corners on both sides as markers to ensure that the membrane was cut from the same source. The portion displayed in the article is highlighted within a red rectangle.

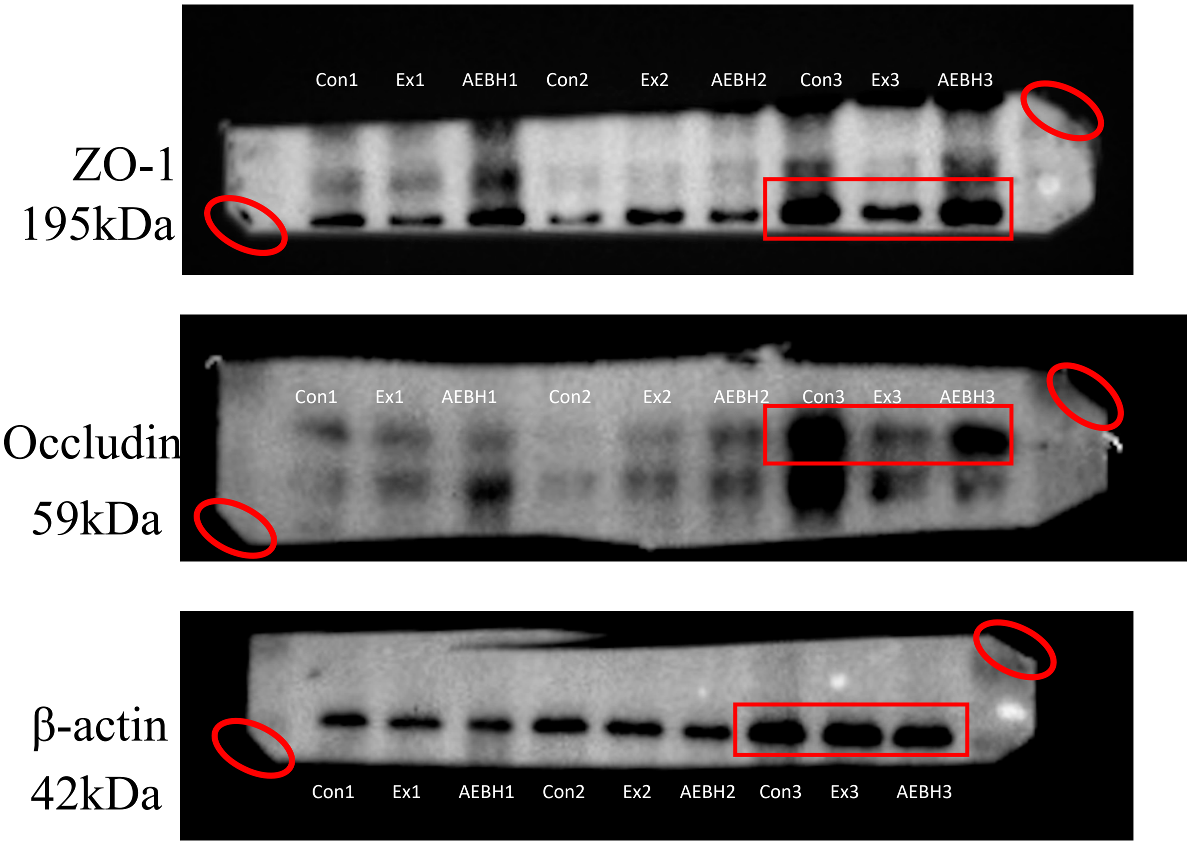

Figure S4. Raw blot images for ZO-1, Occludin protein

Table S1. Ingredient composition of animal feed

Ingredient	Composition
Maize	Grain-based ingredients constitute 80%
Wheat middling	
Wheat	
Medicago sativa	
Bean pulp	
Fish meal	Animal protein constitutes 10%
Chicken meal	
Animal premix	Minor ingredient additives constitute 10%
Wheat gluten	
Calcium bicarbonate	
Rock powder	
Salad oil	
Feed grade sodium chloride	
Feed grade magnesia	
Total	1

Table S2. Macronutrients in animal feed

	Amount
Percentage of crude protein, %	20.8
Percentage of crude lipid, %	5.6

Percentage of carbohydrate, %	58
Energy composition	
Total energy, kcal/kg	3656
Protein energy ratio, %	22.8
Lipid energy ratio, %	13.8
Carbohydrate energy ratio, %	63.4
Total	100%

Table S3. Micronutrients in animal feed

	Amount
Vitamin A, IU/kg	1.49×10^4
Vitamin E, IU/kg	109
Vitamin B_1, mg/kg	17.7
Vitamin B_2, mg/kg	13.7
Vitamin B_6, mg/kg	20.3
Vitamin D_3, IU/kg	1.73×10^3
Vitamin C, mg/kg	Undetected (< 0.044)
Magnesium, %	0.194
Potassium, %	0.624
Sodium, %	0.281
Calcium, g/kg	11.3
Phosphorus, g/kg	7.0
Iron, mg/kg	277
Manganese, mg/kg	196
Copper, mg/kg	13.8
Zinc, mg/kg	134
Selenium, mg/kg	0.22
